# Radiation Exposure and Safety Considerations in Interventional Radiology: Comparison of a Twin Robotic X-ray System to a Conventional Angiography System

**DOI:** 10.3390/jcm13102732

**Published:** 2024-05-07

**Authors:** Christer Ruff, Sasan Partovi, Isabella Strobel, Stella Kaleth, Klaus Herz, Konstantin Nikolaou, Abraham Levitin, Levester Kirksey, Roland Syha, Christoph Artzner, Gerd Grözinger

**Affiliations:** 1 Department of Diagnostic and Interventional Neuroradiology, University Hospital Tuebingen, Hoppe-Seyler-Strasse 3, 72076 Tuebingen, Germany; 2 Department of Diagnostic and Interventional Radiology, University Hospital Tuebingen, Hoppe-Seyler-Strasse 3, 72076 Tuebingen, Germany; 3 Interventional Radiology, Cleveland Clinic Main Campus, Cleveland, OH 44195, USA; 4 Department of Radiation Protection, University Hospital Tuebingen, Auf der Morgenstelle 24, 72076 Tuebingen, Germany; 5 Department of Nuclear Medicine, University Hospital of Würzburg, Oberduerrbacher Straße 6, 97080 Wuerzburg, Germany; 6 Vascular Surgery, Cleveland Clinic Main Campus, Cleveland, OH 44195, USA; 7 Department of Diagnostic and Interventional Radiology, Prosper Hospital, Muehlenstraße 27, 45659 Recklinghausen, Germany; 8 Department of Diagnostic and Interventional Radiology, Diakonie Klinikum Stuttgart, Rosenbergstraße 38, 70176 Stuttgart, Germany

**Keywords:** radiation dose, Alderson RANDO phantom, angiography system, twin robotic X-ray system

## Abstract

**Background/Objectives:** To evaluate radiation exposure in standard interventional radiology procedures using a twin robotic X-ray system compared to a state-of-the-art conventional angiography system. **Methods:** Standard interventional radiology procedures (port implantation, SIRT, and pelvic angiography) were simulated using an anthropomorphic Alderson RANDO phantom (Alderson Research Laboratories Inc. Stamford, CT, USA) on an above-the-table twin robotic X-ray scanner (Multitom Rax, Siemens Healthineers, Forchheim, Germany) and a conventional below-the-table angiography system (Artis Zeego, Siemens Healthineers, Forchheim, Germany). The phantom’s radiation exposure (representing the potential patient on the procedure table) was measured with thermoluminescent dosimeters. Height-dependent dose curves were generated for examiners and radiation technologists in representative positions using a RaySafe X2 system (RaySafe, Billdal, Sweden). **Results:** For all scenarios, the device-specific dose distribution differs depending on the imaging chain, with specific advantages and disadvantages. Radiation exposure for the patient is significantly increased when using the Multitom Rax for pelvic angiography compared to the Artis Zeego, which is evident in the dose progression through the phantom’s body as well as in the organ-related radiation exposure. In line with these findings, there is an increased radiation exposure for the performing proceduralist, especially at eye level, which can be significantly minimized by using protective equipment (*p* < 0.001). **Conclusions:** In this study, the state-of-the-art conventional below-the-table angiography system is associated with lower radiation dose exposures for both the patient and the interventional radiology physician compared to an above-the-table twin robotic X-ray system for pelvic angiographies. However, in other clinical scenarios (port implantation or SIRT), both devices are suitable options with acceptable radiation exposure.

## 1. Introduction

The rising numbers of fluoroscopy-guided interventional procedures result in an increased radiation burden for both patients and involved medical staff. Complex endovascular and interventional oncologic therapies within the abdomen and pelvis mandate for continuous technology development to decrease radiation exposure [1,2,3]. In combination with a trend towards obesity in different patient populations, radiation safety concerns are gaining increasing importance [4]. Hence, radiation protection (RP) needs to be used meticulously during fluoroscopically guided procedures (FGP) to accomplish as low as reasonably achievable radiation exposure levels [5]. Of note, Li et al. reported alarmingly high effective doses in a substantial number of patients who had one or more FGP [6]. A combination of training, protective equipment, and technological innovations have the potential to reduce radiation exposure in the interventional radiology suite [7,8,9,10,11]. The precautionary guidelines of the International Atomic Energy Agency (IAEA) should be used when working with radiation.

While conventional angiography systems have been established for decades, hybrid X-ray systems with angiography capabilities were recently introduced to the clinical arena. For this setup of hybrid X-ray systems, several studies investigated radiation doses of cardiac interventions, though limited data exist for fluoroscopy-guided interventions in the abdomen and pelvis performed by interventional radiology physicians. The, at the time of the study, recently released twin robotic X-ray system Multitom Rax (Siemens Healthineers, Forchheim, Germany) is a conventional X-ray system with an integrated detector for static, dynamic, and 3D imaging that offers the opportunity of fluoroscopy-guided interventional procedures. Multitom Rax is a device intended to visualize anatomical structures by converting an X-ray pattern into a visible image. The system has a broad variety of medical applications, including gastrointestinal examinations, skeletal, thoracic, and lung exposures, as well as examinations of the urogenital tract. However, the unit may also be used in myelography, venography, arthrography, interventional radiology, digital angiography, and digital subtraction angiography (DSA). The aim of this study was to compare radiation exposure between a state-of-the-art conventional angiography system with a below-the-table angiography setup (Axiom Artis Zeego, Siemens Healthineers, Forchheim, Germany) versus the twin robotic X-ray system with an above-the-table X-ray tube (Multitom Rax, Siemens Healthineers, Forchheim, Germany). In this study, the new angiography system was investigated in simulated body regions of the thorax, upper abdomen, and pelvis, in which endovascular and minimal invasive interventional oncologic procedures are being performed.

## 2. Materials and Methods

### 2.1. Phantom Study

An anthropomorphic, hermaphrodite male phantom (Alderson RANDO phantom, Alderson Research Laboratories Inc., Stanford, CT, USA) was utilized for experimental dose exposure measurements for patients and performing medical staff applying the manufacturer’s default settings for the respective angiography system and region of interest (Table 1). Simulated representative procedures in this study are a diagnostic angiogram of the pelvis, a selective internal radiation therapy (SIRT) procedure of the upper abdomen/hepatic region, and a ported venous catheter placement in the chest. The same image field was selected on both devices for the respective examination based on the DSA image. Dose area products (DAP) are an established proxy for the effective radiation dose and were measured intrinsically by both systems [12,13]. The accuracy of the systems’ intrinsic DAP measurements is confirmed by the institute’s radiation physicists every three months via standardized measurements.

### 2.2. Angiography Systems

This study was pursued on an Axiom Artis Zeego (Artis Zeego, Siemens Healthineers, Forchheim, Germany) flat panel detector system (FPD) and above-the-table twin robotic X-ray system (Multitom Rax, Siemens Healthineers, Forchheim, Germany); Supplemental Figure S1. Both X-ray systems are equipped with an automatic exposure control mechanism.

The Axiom Artis Zeego is a monoplane FPD-based system in which a scintillator layer composed of Cesium-iodide (CsI) crystals converts X-ray photons to light photons, which are detected by photodiodes. The system consists of a flat panel detector of 30 cm × 40 cm and a rotating anode X-ray tube mounted on a C-arm, which is by itself attached to a 6-axis robot. It is capable of pulsed fluoroscopy up to a frame rate of 30 pulses/s, spot film mode, and three magnification modes. The system uses a dedicated automatic dose control and automatic spectral beam selection process during fluoroscopy. The X-ray tube is the Megalix Cat125/15/40/80 three-focus high-performance X-ray tube assembly. The focal spot sizes are 0.3/0.6/1.0 mm with power ratings of 15/40/80 kW, respectively, and it operates in the range 50 to 125 kV. Digital pulsed fluoroscopy (low-dose imaging) ranges from 0.5 to 30 frames per second (fps) in a 1k/12-bit matrix with real-time filtering to accomplish noise reduction including motion detection. Digital radiography and digital subtraction angiography are provided at frame rates ranging from 0.5 to 7.5 fps in original matrix size.

The twin robotic system Multitom Rax is equipped with two motor-driven telescopic arms carrying a flat panel detector and the X-ray tube, respectively. Both arms are mounted on ceiling rails and can move independently to predetermined positions within the radiography suite for 2D and fluoroscopic imaging with two rotational and three translational degrees of freedom. The monoplane system includes a ceiling-mounted built-in CsI scintillator detector (Trixell Pixium 4343-F4) of 43 cm × 43 cm and three magnification views. It is capable of pulsed fluoroscopy up to a frame rate of 30 pulses/s. The X-ray tube assembly is the Optitop 150/40/80HC-100, which is operated in a pulsed mode with a selectable nominal focal spot size (IEC 60336) of either 0.6 (0.9 mm × 1.3 mm) or 1.0 (1.4 mm × 2.0 mm). The X-ray tube operates at tube voltages in the range of 40 to 150 kV, with resulting tube current–time products between 0.5 mAs and 800 mAs.

### 2.3. Experimental Dose Exposure Measurements for Patients

The Alderson RANDO phantom was equipped with calibrated thermoluminescent dosimeters (TLD, TLD-100H, Bicon-Harshow, Radiation Measurement Products, Cleveland, Ohio). For calibration, TLDs were annihilated at high temperature for several hours. Thus, all electrons from the adhesion sites fall back into the valence band. The calibration factors with the unit µGy/nC are determined by irradiating the TLDs with 10 mGy and then reading them out twice. The double readout is based on the assumption that a residual excitation is retained in the TLDs. Subsequently, TLDs can be used for dosimetry. These were positioned in the phantom to assess the individual organ doses (Supplemental Figure S2). The average times of fluoroscopy (FL) and digital subtraction angiography (DSA) of 30 consecutively performed examinations on the Axiom Artis Zeego system were used for each simulated angiographic procedure (Table 2). FL times for port implantation, SIRT, and pelvic angiography were 1 min 16 s, 13 min and 42 s, and 4 min and 30 s, respectively. DSA times for port implantation, SIRT, and pelvic angiography were a single image, 1 s, and 60 s, respectively. Due to intrinsic measurement errors of TLDs and to map a larger section of organs, redundant datapoints were acquired for each organ with the exception of the urinary bladder and the gallbladder. All measurements were repeated three times with the aim of reducing measurement errors. Doses of larger organs (i.e., rectum) were assessed by the mean of the organ’s TLDs. The uterus and ovaries were used to measure female-specific radiation exposure and the prostate was used for male-specific radiation exposure. Due to the male phantom, no specific measurements of the breast dose were carried out. The anterodorsal dose profile was measured in the center of the X-ray field. For ported venous catheter placements, TLDs were placed in the position of the thyroid gland (n = 4), esophagus (n = 3), spinal cord (n = 3), skin (entry and exit dose, each n = 3), and heart (n = 4). For the SIRT procedure, TLDs were placed in the position of the gallbladder (n = 1), heart (n = 4), spinal cord (n = 1), and skin level (entry n = 8 and exit dose n = 11). For the diagnostic angiography of the pelvis, TLDs were placed in the position of the rectum (n = 11), uterus (n = 5), ovaries (n = 4), prostatic gland (n = 4), urinary bladder (n = 1), and skin level (entry n = 5 and exit dose n = 7).

The evaluation of the irradiated TLDs was performed using a TLD reader (Model Harshaw TLD^TM^ 5500 TLD Reader, ThermoFischer Scientific, Waltham, MA, USA) within 24 h after radiation exposure to avoid systematic errors. The readout TLD values in nanocoulombs were multiplied by an individual calibration factor, which was defined by means of parallel exposure of TLDs with a known radiation dose using 102 kV and 10 mAs for 34 milliseconds at a source-to-skin distance (SSD) of 100 cm (Philips Optimus65, Philips Medical Systems, PC Best, Amsterdam, The Netherlands). To minimize the Heel effect, wire markers in the field were avoided and all exposures were conducted in the same position with respect to the orientation of the X-ray tube.

### 2.4. Experimental Dose Exposure Measurements for Procedural Staff

**Figure 1:**
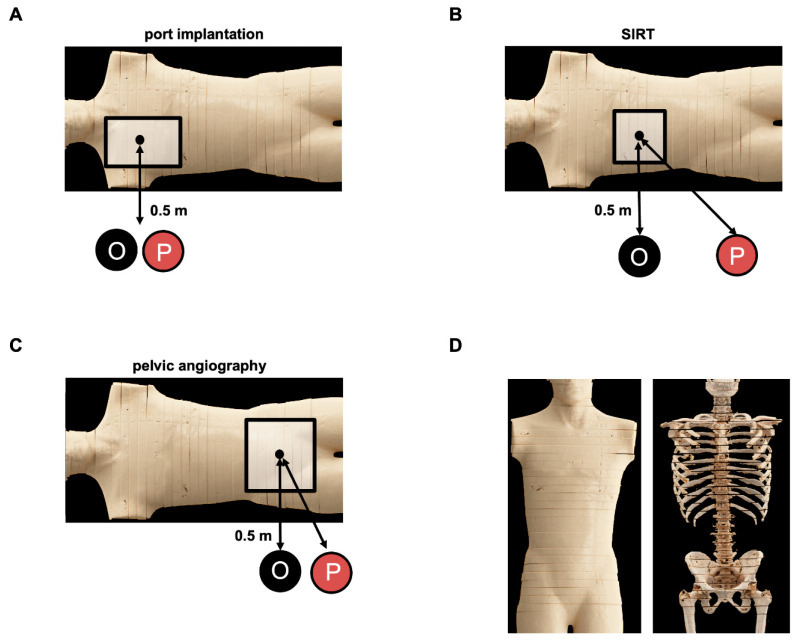
Experimental setup of dose exposure measurements for both the medical staff and the patient using the below-the-table angiography system Artis Zeego or the above-the-table twin robotic X-ray system Multitom Rax. Simulated body regions cover the chest, upper abdomen, and pelvis with the corresponding representative procedures, being ported venous catheter placement (**A**), SIRT procedure (**B**), and diagnostic pelvic angiography (**C**). An anthropomorphic Alderson RANDO phantom was used for the respective experiments, partially displayed with volume rendering technique (VRT) using a computed tomography (**D**). P = proceduralist’s position, O = orthogonal measurement location to radiation source and table. SIRT = selective internal radiation therapy.

The setup for the experimental dose exposure measurements for procedural staff is demonstrated in Figure 1. Experimental dose measurements for procedural staff were acquired using the same setup as for patients’ dose measurements. Height-dependent dose curves were generated for the examiners and the medical technical assistants in representative positions using a RaySafe X2 system (RaySafe, Billdal, Sweden). Steady-state radiation dose curves were acquired from 55 cm above the floor to the eye height of a standard person (165 cm), with measurements every 10 cm. Scatter radiation of DSA and FL was measured for 10 s for each position. Measurements were repeated three times for eye level (165 cm), table level (95 cm), as well as the level of the back and front of the Alderson RANDO phantom. The last three locations correspond to sites with the known highest level of scattered radiation. All other levels were measured one time due to acquisition of robust data for eye level. Measurements were performed with and without protective equipment. For the Multitom Rax system, these included a ceiling-suspended lead shield for the pelvis and upper abdomen. Additional MAVIG lead pads (MAVIG, Munich, Germany) were placed next to the femoral arterial vascular access site for upper abdomen procedures.

### 2.5. Statistical Analysis

Statistical analysis was performed using JMP 14.2 (SAS Institute Inc., Cary, NC, USA) and SPSS (release 26 for Windows; SPSS, Chicago, IL, USA). A *p*-value equal to or less than 0.05 was considered statistically significant. Due to multiple testing, Bonferroni corrected *p*-values were determined. Results of the radiation dose measurements were expressed as mean and standard deviation. Cohen’s kappa coefficient d effect sizes were calculated and interpreted according to the following criteria: non to slight |d| = 0.01–0.2; fair |d| = 0.21–0.40; moderate |d| = 0.41–0.60; substantially large |d| = 0.41–0.60; almost perfect agreement |d| = 0.81–1.00 [14].

## 3. Results

### 3.1. Patient Radiation Exposure in Relation to the Anatomical Localization and Procedure

**Figure 2:**
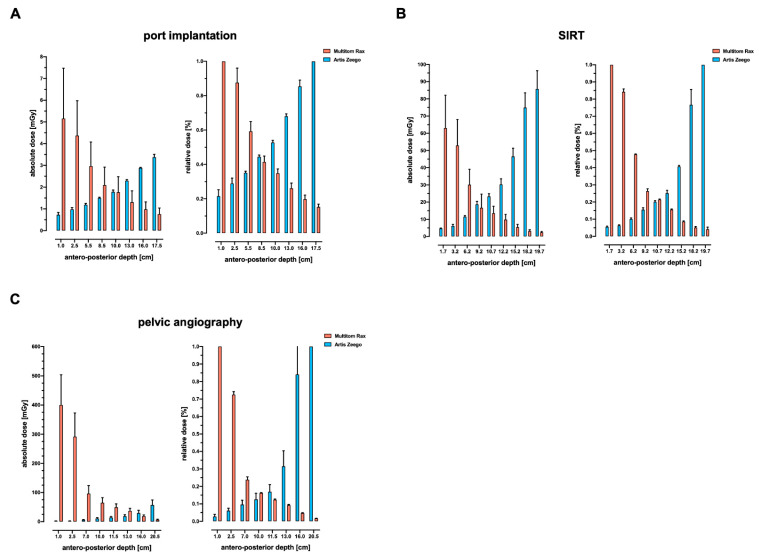
Antero–posterior dose profiles of a representative slice in the center of the beam path using an anthropomorphic Alderson RANDO phantom equipped with thermoluminescent dosimeters (TLD). Respective body regions and simulated examinations include ported venous catheter placement (**A**), SIRT procedure (**B**), and diagnostic pelvic angiography (**C**). Default settings of the below-the-table angiography system Artis Zeego and the above-the-table twin robotic X-ray system Multitom Rax were applied. port = ported venous catheter placement, SIRT = selective internal radiation therapy.

Figure 2 depicts the anterior–posterior depth–dose profiles of both angiography systems using the Alderson RANDO phantom in the center of the X-ray beam path. The simulated body regions cover the thorax (ported venous catheter placement, Figure 2A), upper abdomen (SIRT procedure, Figure 2B), and the pelvis (invasive angiography of the pelvis, Figure 2C). The graphs show typical patterns of exponential decay after entering the phantom.

**Figure 3:**
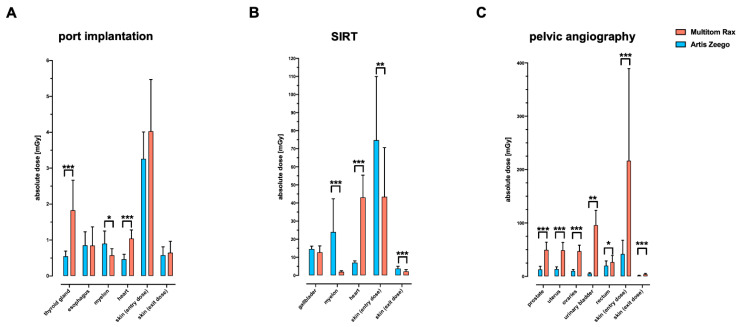
Absolute organ-related radiation exposure (mGy) using an anthropomorphic Alderson RANDO phantom equipped with thermoluminescent dosimeters (TLD). Respective body regions and simulated examinations include ported venous catheter placement (**A**), SIRT procedure (**B**), and diagnostic pelvic angiography (**C**). Default settings of the below-the-table angiography system Artis Zeego and the above-the-table twin robotic X-ray system Multitom Rax were applied. port = ported venous catheter placement, SIRT = selective internal radiation therapy. Significant differences are indicated with asterisks (* *p* < 0.05, ** *p* < 0.01, *** *p* < 0.001).

For ported venous catheter placements, the data reveal a comparable radiation exposure in the depth–dose profile comparing Artis Zeego to Multitom Rax (1.9 ± 1.0 vs. 2.2 ± 1.8 mGy, *p* = 0.412); Figure 2A. The above-the-table twin robotic X-ray system Multitom Rax (at low level radiation exposure levels) demonstrates a significantly higher radiation exposure for the thyroid gland (0.5 ± 0.1 vs. 1.8 ± 0.8 mGy, *p* = < 0.001) and heart (0.5 ± 0.1 vs. 1.0 ± 0.2 mGy, *p* < 0.001); Figure 3A and Table 2. The Artis Zeego, however, led to a significantly higher radiation exposure for the spinal cord in comparison to the Multitom Rax (0.9 ± 0.3 vs. 0.6 ± 0.2, *p* = 0.02).

The simulated SIRT procedure revealed significantly higher radiation exposure values to the heart using the Multitom Rax in comparison to the Artis Zeego (43.1 ± 12.3 mGy vs. 7.1 ± 1.0 mGy, *p* < 0.001). However, the Artis Zeego led to a significantly higher radiation exposure at the skin entry level (71.2 ± 38 vs. 43.5 ± 27.1 mGy, *p* = 0.004) as well as skin exit site (3.8 ± 1.2 vs. 2.2 ± 1.0 mGy, *p* < 0.001); Figure 3B and Table 2.

For pelvic angiography procedures, the above-the-table twin robotic X-ray system Multitom Rax was associated with a significantly higher radiation dose exposure compared to the conventional angiography system Artis Zeego both in the depth–dose profile (120.5 ± 144.1 mGy vs. 18 ± 18.2 mGy, *p* = 0.002) and the majority of organ-related dose measurements, as demonstrated in Figure 2C and Figure 3C. The Multitom Rax caused a significantly increased organ-related radiation exposure for this procedure in the area of the prostatic gland (49.8 ± 14.4 vs. 13.1 ± 5.7 mGy, *p* < 0.001), the uterus (48.9 ± 14.6 vs. 13.9 ± 4.3 mGy, *p* < 0.001), the ovaries (47.5 ± 11 vs. 10.3 ± 2.8 mGy, *p* < 0.001), the urinary bladder (96 ± 27.8 vs. 5.6 ± 1.5 mGy, *p* = 0.005), the skin entry (42 ± 25.9 vs. 216.6 ± 172.6 mGy, *p* < 0.001), as well as the skin exit site (1.5 ± 0.7 vs. 4.2 ± 1.5 mGy, *p* < 0.001). For further details, see Figure 3C and Table 2.

### 3.2. Radiation Exposure during Ported Venous Catheter Placements

**Figure 4:**
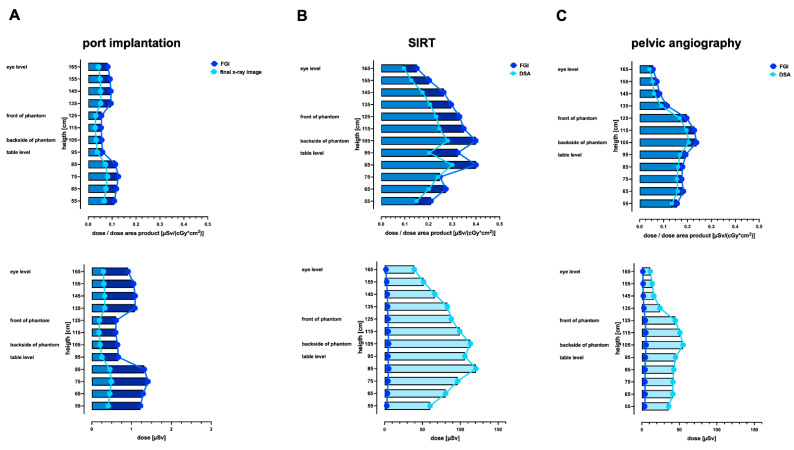
Comparison of height profiles of the scattered radiation orthogonal to the radiation center during ported venous catheter placement, SIRT procedure, and pelvic angiography using the below-the-table angiography system Artis Zeego. Data are demonstrating scattered radiation as a ratio of dose and dose area product (μSV/(cGy*cm^2^)) as well as in absolute dose numbers (μSV) orthogonal to the radiation center of ported venous catheter placement (**A**), SIRT procedure (**B**), and pelvic angiography (**C**). Default settings were applied. FGI = fluoroscopy-guided imaging, DSA = digital subtraction angiography, port = ported venous catheter placement, SIRT = selective internal radiation therapy.

**Figure 5:**
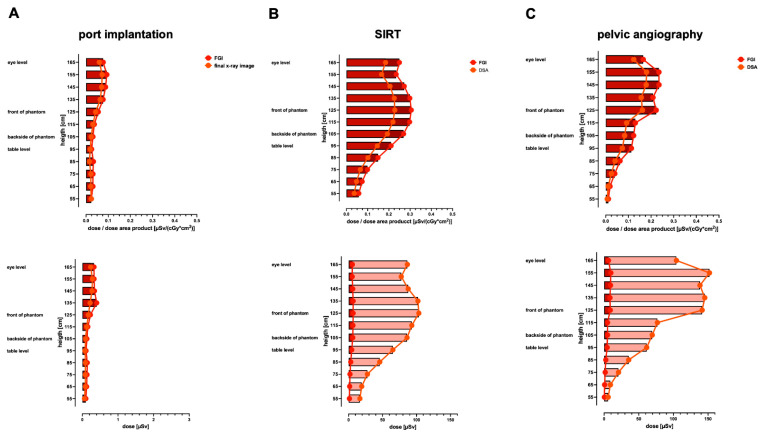
Comparison of height profiles of the scattered radiation orthogonal to the radiation center during ported venous catheter placement, SIRT procedure, and pelvic angiography using the above-the-table twin robotic X-ray system Multitom Rax. Data demonstrate scattered radiation as a ratio of dose and dose area product (μSV/(cGy*cm^2^)) as well as in absolute dose numbers (μSV) orthogonal to the radiation center of the ported venous catheter placement (**A**), SIRT procedure (**B**), and pelvic angiography (**C**). Default settings were applied. FGI = fluoroscopy-guided imaging, DSA = digital subtraction angiography, port = ported venous catheter placement, SIRT = selective internal radiation therapy.

The above-the-table twin robotic X-ray system Multitom Rax showed significantly lower scattered radiation in FGI and DSA compared to the conventional angiography system Artis Zeego (Figure 4A and Figure 5A). The absolute measured scattered radiation (μSV) orthogonal to the radiation center of FGI at eye level for Multitom Rax vs. Artis Zeego is 0.327 ± 0.003 vs. 0.914 ± 0.009 (*p* < 0.001); at the table level, it is 0.104 ± 0.084 vs. 0.667 ± 0.005 (*p* < 0.001); and, at the phantom’s front (entry site), it is for the Multitom Rax system 0.225 ± 0.004. At comparable heights, the absolute measured scattered radiation (µSv) for the final X-ray image for Multitom Rax vs. Artis Zeego are 0.232 ± 0.003 vs. 0.294 ± 0.021 (*p* = 0.008), 0.256 ± 0.016 vs. 0.072 (*p* < 0.001), and 0.167 ± 0.002, respectively.

### 3.3. Radiation Exposure during the SIRT Procedure

A comparison of the measured dose curves between the Multitom Rax and Artis Zeego orthogonal to the radiation center demonstrates differences for upper abdominal angiography for the SIRT procedure (Figure 4B and Figure 5B). The absolute measured scattered radiation (μSV) orthogonal to the radiation center of FGI at eye level is for Multitom Rax vs. Artis Zeego 5.604 ± 0.655 vs. 2.053 ± 0.007 (*p* < 0.001), at table level 4.609 ± 0.103 vs. 4.342 ± 0.033 (*p* = 0.013), and, at the phantom’s front (entry site) for the Multitom Rax system, 6.697 ± 0.005. At comparable heights, the absolute measured scattered radiation for DSA comparing the Multitom Rax system vs. Artis Zeego is 86.5 ± 5.906 vs. 39.213 ± 2.552 (*p* < 0.001), 65.20 ± 0.595 vs. 106.107 ± 11.655 (*p* = 0.004), and 103.433 ± 1.656. Consistent with the antero-posterior dose profiles (Figure 2B) and measured organ doses (Figure 3B), especially the entry dose at the skin level, the scattered radiation dose at table height and the backside is increased at these locations using the Artis Zeego (Figure 4B). In contrast, radiation exposure at eye level is higher with the Multitom Rax system than with the Artis Zeego due to the above-the-table tube.

### 3.4. Radiation Exposure during Invasive Angiography of the Pelvis

The comparison of the measured dose curves for scattered radiation between the Multitom Rax and Artis Zeego for diagnostic pelvic angiograph reveals the most significant differences of all three examined procedures with the Multitom Rax system being associated with significantly higher radiation exposure compared to the Artis Zeego system (Figure 4C and Figure 5C). The absolute measured scattered radiation (μSV) of the Multitom Rax compared to the Artis Zeego orthogonal to the radiation center of FGI at eye level is 6.271 ± 0.004 vs. 1.368 ± 0.041 (*p* < 0.001), at table level is 4.413 ± 0.099 vs. 4.705 ± 0.041 (*p* = 0.032), and, at the phantom’s front (entry site) for the Multitom Rax system, is 8.905 ± 0.096. At comparable heights, the absolute measured scattered radiation for DSA is 104.4 ± 3.291 vs. 11.235 ± 2.08 (*p* < 0.001), 61.543 ± 2.438 vs. 45.055 ± 0.728 (*p* = 0.003), and 141.47 ± 6.017 for the Multitom Rax system vs. Artis Zeego. These differences in radiation exposure are consistent with the antero-posterior dose profiles of a representative slice of the anthropomorphic Alderson RANDO phantom in the center of the beam path (Figure 2C).

### 3.5. Radiation Exposure for the Performing Proceduralist during SIRT and Invasive Angiography of the Pelvis Using the Multitom Rax System

The Multitom Rax is associated with particularly high scattered radiation exposure of the upper body region for the performing proceduralist due to the above-the-table radiation source and the resulting high scattered radiation at the patient’s entry point at the front. Due to the significantly higher radiation exposure, especially during pelvic angiography (Figure 5C), the extent to which protective measures can reduce this exposure for the performing proceduralist were investigated in a further step in the typical standing position. For the SIRT procedure, a ceiling-suspended screen on its own and in combination with X-ray protective lead pads placed immediately proximal to the femoral arterial vascular access site were tested. For pelvic angiography, only a ceiling-suspended screen is available for the proceduralist as a protective measure and this was tested in the current study.

For the SIRT procedure, the absolute measured scattered radiation (μSV) for the Multitom Rax system at the position of the performing proceduralist of FGI at eye level without X-ray protection is 2.853 vs. 0.032 with a ceiling-suspended screen (*p* < 0.001), at the phantom’s front 3.544 vs. 0.131 with a ceiling-suspended screen (*p* < 0.001), and, at table level, it is 1.46 vs. 0.168 (*p* < 0.001). At comparable heights, the absolute measured scattered radiation for DSA for the Multitom Rax system at eye level without X-ray protection is 41.827 vs. 0.238 with a ceiling-suspended screen (*p* < 0.001), at the phantom’s front it is 61.03 vs. 1.675 (*p* < 0.001), and, at table level, it is 20.83 vs. 1.748 (*p* < 0.001). For the SIRT procedures, the measured scattered radiation is further reduced by up to 37.1 to 55.5% for FGI and up to 22–62.9% for DSA when using X-ray protective lead pads placed immediately proximal to the femoral vascular arterial access site compared to the sole use of the ceiling-suspended screen; Table 3.

**Figure 6:**
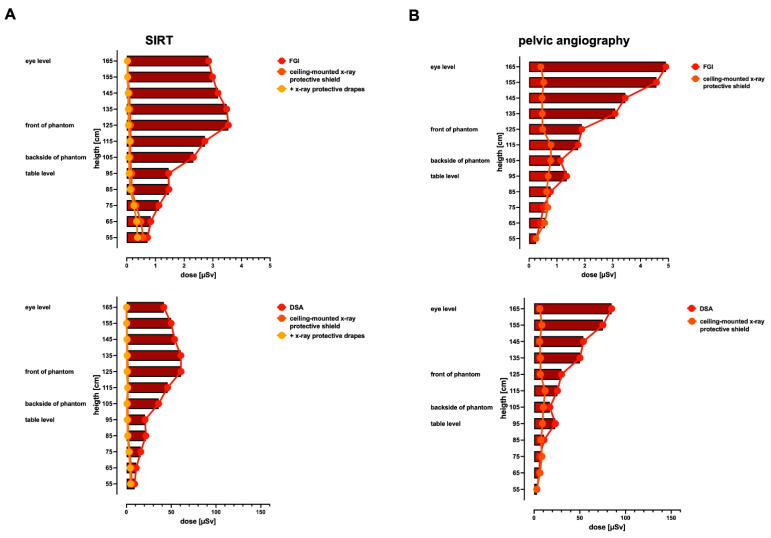
Height profiles of the scattered radiation in the position of the proceduralist during SIRT and pelvic angiography using the above-the-table twin robotic X-ray system Multitom Rax with radiation protection. Data demonstrate scattered radiation of FGI and DSA for SIRT procedure (**A**) and pelvic angiography (**B**) without and with radiation protection, i.e., ceiling-suspended screens on their own and in combination with X-ray protective drapes for the SIRT procedure. Default settings were applied. FGI = fluoroscopy-guided imaging, DSA = digital subtraction angiography, port = ported venous catheter placement, SIRT = selective internal radiation therapy.

**Figure 7:**
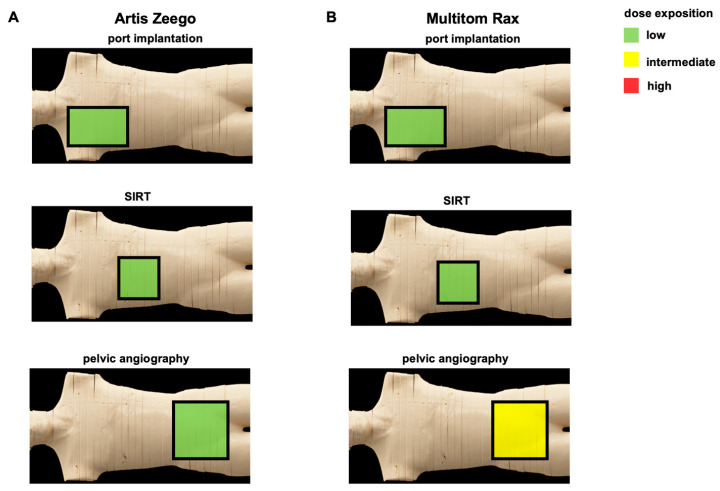
Simplified overview of results for tested procedures using Artis Zeego or Multitom Rax. Simulated body regions cover the chest, upper abdomen, and pelvis with the corresponding representative procedures, being ported venous catheter placement, SIRT procedure, and diagnostic pelvic angiography using Artis Zeego (**A**) or Multitom Rax (**B**). port = ported venous catheter placement, SIRT = selective internal radiation therapy.

For pelvic angiography, the absolute measured scattered radiation (μSV) for the Multitom Rax system at the position of the performing proceduralist of FGI at eye level without X-ray protection is 4.911 vs. 0.42 with a ceiling-suspended screen (*p* < 0.001); at the phantom’s front, it is 1.893 vs. 0.489 (*p* < 0.001); and, at table level, it is 1.352 vs. 0.689 (*p* < 0.001). The absolute measured scattered radiation for the Multitom Rax system at comparable heights for DSA is 84.82 vs. 6.295 (*p* < 0.001), 30.06 vs. 6.935 (*p* < 0.001), and 23.26 vs. 9.2 (*p* = 0.017). In summary, the radiation exposure for the performing proceduralist is significantly reduced for both procedures using protective measures, especially at eye level (Figure 6A,B, Table 3). Based on the results of this study, a simplified depiction of overall results for port implantation, SIRT, and pelvic angiography are given in Figure 7.

## 4. Discussion

In this study, the comparison of the state-of-the-art conventional below-the-table angiography system Artis Zeego to the above-the-table twin robotic X-ray scanner Multitiom Rax revealed significant differences with regard to radiation exposure during pelvic angiography procedures for both the patient and performing proceduralist, favoring the conventional angiography system. For port implantations and SIRT procedures, the results showed a mixed picture, favoring the Artis Zeego or the Multitom Rax depending on the simulated body region. Using appropriate X-ray protection in combination with the above-the-table X-ray scanner, the radiation exposure for the performing proceduralist could be significantly reduced.

Proper assessment of radiation exposure is of utmost importance in daily clinical practice since it harbors risk for both the patient and medical staff. The steadily increasing use of FGIs in daily clinical practice is related to the benefits and safety profile of these minimal invasive procedures [7]. However, it is well known that FGI procedures can accumulate high radiation doses to patients and that these doses can result in radiation-induced skin injuries of varying degrees [15,16]. All angiographic procedures should have, therefore, a goal to reduce radiation exposure as low as reasonably achievable (ALARA) [5] and, therefore, dose optimization and reduction for FGI is highly relevant. In the angiography suite, medical staff and the operator are exposed to lower single but higher cumulative radiation doses than the patient. An inclusive radiation safety program protects the patient and entire medical staff. Since radiation exposure is a crucial challenge in interventional radiology, the Society of Interventional Radiology (SIR) and the Cardiovascular and Interventional Radiology Society of Europe (CIRSE) have published joint guidelines on occupational radiation protection in Interventional Radiology, highlighting the need for appropriate education, training of the staff involved in FGP, and the availability of appropriate protection tools and equipment as well as the importance of technological advancements [17,18].

In this study for both tested angiography systems, the location of the highest dose of scattered radiation is at different locations, owing to the inherent nature of the devices, since the point of origin of the highest scattered radiation is typically at the patient’s entry site. This research study demonstrates that the patient’s radiation exposure for pelvic angiography procedures using the Multitom Rax is significantly higher compared to the Artis Zeego. Even if the increased scattered radiation for the medical staff can be reduced by suitable protective equipment, the significantly increased radiation exposure for the patient needs to be taken into consideration. This applies to the SIRT procedure as well, especially for scattered radiation at eye level, which can be significantly reduced by protective measures for the performing proceduralist. A further reduction in the scattered radiation would presumably still be feasible by improved positioning of the lead shield with the attached lead lamellae of the Multitom Rax system. In the current study protocol, there are small gaps between the lead shield’s attached lead lamellae and the surface of the RANDO Alderson phantom. This results in the escape of small quantities of scattered radiation in the direction of the performing proceduralist.

This study has several limitations. First, the manufacturer’s recommended default settings for each angiography system were applied. Therefore, in this preliminary study, the different procedures were not compared based on identical settings. The performance of both systems may be impacted by electronic and software configurations. Second, the effect of radiation safety glasses and equipment for the performing proceduralist were not separately tested for either angiography system using the existing setup. Third, when comparing two different angiography systems, the question arises whether diagnostic images with similar quality were obtained. As the aim of this study was primarily the comparison of radiation exposure as a safety consideration during first clinical implementations, a comparison of the image quality based on the default settings of each angiography system was not pursued in this investigation. Accordingly, further optimization of the dose through adapted protocols while also considering the image quality would be conceivable.

## 5. Conclusions

The tested state-of-the-art conventional below-the-table angiography system offers superior radiation protection, both for the patient and the performing proceduralist, compared to an above-the-table twin robotic X-ray system for pelvic angiography procedures. Therefore, the conventional angiography system should be preferred for pelvic angiographies. The differences in the conventional below-the-table angiography system compared to the above-the-table twin robotic X-ray system were less pronounced and mixed for port implantation and SIRT, favoring the Artis Zeego or the Multitom Rax depending on the simulated body region. Both devices therefore represent an alternative for this type of procedure.

## Figures and Tables

**Table 1:** Manufacturer’s default settings and acquisition parameters of the below-the-table angiography system Axiom Artis Zeego and the above-the-table twin robotic scanner Multitom Rax. FGI = fluoroscopy-guided imaging, DSA = digital subtraction angiography.

Characteristics	FGI		DSA	
Port Implantation	Artis Zeego	Multitom Rax	Artis Zeego	Multitom Rax
Tube voltage (kV)	67.7	73	70	67.5
Cu (mm)	0.2	0.2	0	0
Frames/second	7.5	10	final, single image	final, single image
Acquisition time for experimental dose exposure measurements for patients (minutes)	1:16	1:16	single image	single image
Acquisition time for experimental dose exposure measurements for height profiles (minutes)	00:10	00:10	00:10	00:10
**SIRT**				
Tube voltage (kV)	67.7	73	68	67.5
Cu (mm)	0.2	0.2	0.6/0.3	0
Frames/second	7.5	10	2	2
Acquisition time for experimental dose exposure measurement for patients (minutes)	13: 42	13: 42	00:01	00:01
Acquisition time for experimental dose exposure measurements for height profiles (minutes)	00:10	00:10	00:10	00:10
**diagnostic pelvic angiography**				
Tube voltage (kV)	65	73	66.4	67.5
Cu (mm)	0.2	0.2	0.3	0
Frames/second	7.5	10	2	2
Acquisition time for experimental dose exposure measurement for patients (minutes)	4:30	4:30	01:00	01:00
Acquisition time for experimental dose exposure measurements for height profiles (minutes)	00:10	00:10	00:10	00:10

**Table 2:** Experimental dose exposure measurements for patients using thermoluminescent dosimeters (TLDs). The average of FGI and DSA of 30 consecutive examinations of ported venous catheter placements, SIRT procedures, and diagnostic pelvic angiographies performed on the below-the table angiography system Artis Zeego were used as standard examination time for patient-related experimental dose exposure measurements. Data are means +/− standard deviation (SD). In bold are all significant *p*-values < 0.05. FGI = fluoroscopy-guided imaging, DSA = digital subtraction angiography, SIRT = selective internal radiation therapy.

Location	Artis Zeego Means +/− SD (mGy)	Multitom Rax Means +/− SD (mGy)	*p* Value	Cohen’s D
**port implantation (FGI 1 min 16 s, DSA single image)**				
thyroid gland	0.5 ± 0.1	1.8 ± 0.8	**<0.001**	−2.311
esophagus	0.9 ± 0.4	0.8 ± 0.5	0.979	0.012
spinal cord	0.9 ± 0.3	0.6 ± 0.2	**0.02**	1.116
heart	0.5 ± 0.1	1.0 ± 0.2	**<0.001**	−3.030
skin (entry dose)	3.3 ± 0.7	4.0 ± 1.4	0.101	−0.631
skin (exit dose)	0.6 ± 0.2	0.7 ± 0.3	0.473	−0.284
depth–dose profile	1.9 ± 1.0	2.2 ± 1.8	0.412	−0.199
**angiography for SIRT (FGI 13 min 42 s, DSA 1 s)**				
gallbladder	14.6 ± 1.6	12.8 ± 3.5	0.459	0.669
spinal cord	24 ± 18.3	2.1 ± 0.5	0.107	1.695
heart	7.1 ± 1.0	43.1 ± 12.3	**<0.001**	−3.950
skin (entry dose)	71.2 ± 38	43.5 ± 27.1	**0.004**	0.867
skin (exit dose)	3.8 ± 1.2	2.2 ± 1.0	**<0.001**	1.441
depth–dose profile	33.6 ± 28.7	22.1 ± 23.1	0.117	0.438
**diagnostic pelvic angiography (FGI 4 min 30 s, DSA 60 s)**				
prostate	13.1 ± 5.7	49.8 ± 14.4	**<0.001**	−3.353
uterus	13.9 ± 4.3	48.9 ± 14.6	**<0.001**	−3.256
ovaries	10.3 ± 2.8	47.5 ± 11	**<0.001**	−4.527
urinary bladder	5.6 ± 1.5	96 ± 27.8	**0.005**	−4.600
rectum	20.2 ± 8.8	26.9 ± 12.4	**0.01**	−0.624
skin (entry dose)	42 ± 25.9	216.6 ± 172.6	**<0.001**	−1.552
skin (exit dose)	1.5 ± 0.7	4.2 ± 1.5	**<0.001**	−2.195
depth–dose profile	18 ± 18.2	120.5 ± 144.1	**0.002**	−0.987

**Table 3:** Experimental dose exposure for the performing proceduralist during SIRT procedure and angiography of the pelvis using the above-the-table twin robotic scanner Multitom Rax. The data demonstrate scattered radiation for the performing proceduralist as a ratio of dose and dose area product (μSV/(cGy*cm^2^)) as well as in absolute dose measurements (μSV) for SIRT procedure and pelvic diagnostic angiography without and with protective measures. Exposure time was set at 10 s. Data are means +/− standard deviation (SD). In bold are all significant *p*-values < 0.05. FGI = fluoroscopy-guided imaging, DSA = digital subtraction angiography, SIRT = selective internal radiation therapy.

	SIRT								
Location	(1) No X-ray Protection	(2) Ceiling-Suspended Screen	(3) Ceiling-Suspended Screen + X-ray Protective Drapes	*p* Value (1) vs. (2)	% of Initial Dose (1) vs. (2)	*p* Value (1) vs. (3)	% of Initial Dose (1) vs. (3)	*p* Value (2) vs. (3)	% of Initial Dose (2) vs. (3)
	**FGI (means +/− SD (μSV))**								
eye level	2.853 ± 0.070	0.032 ± 0.004	0.020 ± 0.001	**<0.001**	1.1	**<0.001**	0.1	1	62.9
front of phantom	3.544 ± 0.134	0.131 ± 0.001	0.058 ± 0.002	**<0.001**	3.7	**<0.001**	1.6	0.881	44.5
table level	1.46 ± 0.054	0.168 ± 0.010	0.084 ± 0.005	**<0.001**	11.5	**<0.001**	5.7	0.051	49.7
	**DSA (means +/− SD (μSV))**								
eye level	41.837 ± 0.720	0.238 ± 0.016	0.160 ± 0.008	**<0.001**	0.6	**<0.001**	0.4	1	67
front of phantom	61.03 ± 1.866	1.675 ± 0.053	0.621 ± 0.022	**<0.001**	2.7	**<0.001**	1.0	0.829	37.1
table level	20.83 ± 0.693	1.748 ± 0.027	0.895 ± 0.020	**<0.001**	8.4	**<0.001**	4.2	0.121	51.2
	**diagnostic pelvic angiography**								
**Location**	**(1)** **no x-ray protection**	**(2)** **ceiling-suspended screen**	**N/A**	***p* value** **(1) vs. (2)**	**% of initial dose** **(1) vs. (2)**				
	**FGI (means +/− SD (μSV))**								
eye level	4.911 ± 0.091	0.42 ± 0.008	N/A	**<0.001**	8.6				
front of phantom	1.893 ± 0.004	0.489 ± 0.016	N/A	**<0.001**	25.8				
table level	1.352 ± 0.017	0.689 ± 0.024	N/A	**<0.001**	51				
	**DSA (means +/− SD (μSV))**								
eye level	84.82 ± 1.663	6.295 ± 0.119	N/A	**<0.001**	7.4				
front of phantom	30.06 ± 1.125	6.935 ± 0.239	N/A	**<0.001**	23.1				
table level	23.26 ± 6.178	9.2 ± 0.499	N/A	**<0.017**	39.6				

N/A means not applicable.

## Data Availability

The original contributions presented in the study are included in the article/supplementary material; further inquiries can be directed to the corresponding author on reasonable request.

## Supplementary Materials

The following supporting information can be downloaded at: https://www.mdpi.com/article/10.3390/jcm13102732/s1, Supplemental Figure S1. Image of the Multitom Rax. The Multitom Rax (Siemens Healthineers, Forchheim, Germany) is a twin robotic X-ray system with an above-the-table X-ray tube. The twin robotic system Multitom Rax is equipped with two motor-driven telescopic arms carrying a flat panel detector and the X-ray tube, respectively. Both arms are mounted on ceiling rails and can move independently to predetermined positions within the radiography suite for 2D and fluoroscopic imaging with two rotational and three translational degrees of freedom. Supplemental Figure S2. Experimental setup of the Alderson RANDO phantom equipped with thermoluminescent dosimeters (TLD). An exemplary layer of the Alderson RANDO phantom is depicted for each assessed procedure, namely ported venous catheter placement (A), SIRT procedure (B), and diagnostic pelvic angiography (C) with position of the TLDs including those for the depth dose measurements. SIRT = selective internal radiation therapy.
