# Supplementary figures and images for: Radiation Exposure and Safety Considerations in Interventional Radiology: Comparison of a Twin Robotic X-ray System to a Conventional Angiography System
Source: J Clin Med. 2024 May 7;13(10):2732. doi: 10.3390/jcm13102732 (PMC11122633; doi:10.3390/jcm13102732)

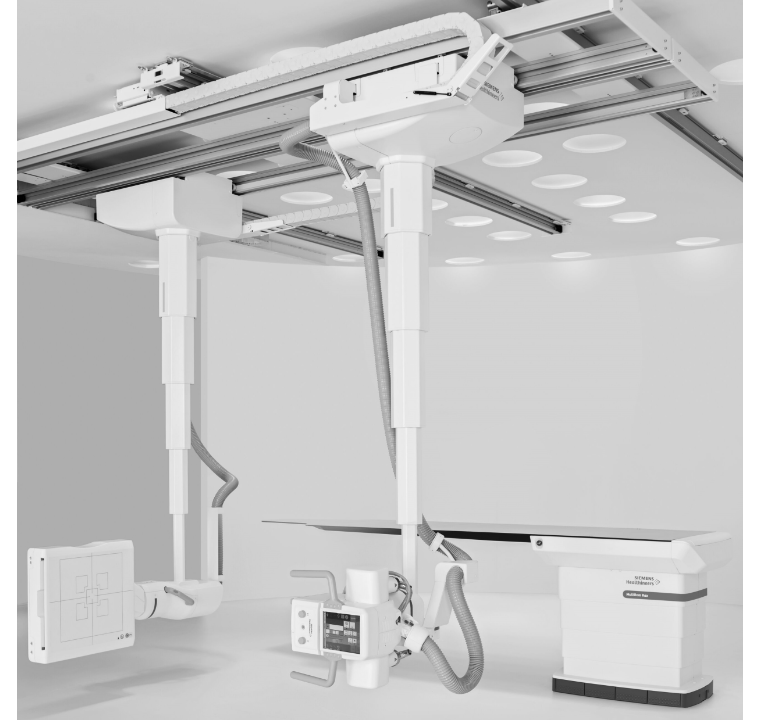

Supplement: Supplementary file 1 [file jcm-13-02732-s001.zip › Supplemental Figure S1.tif]

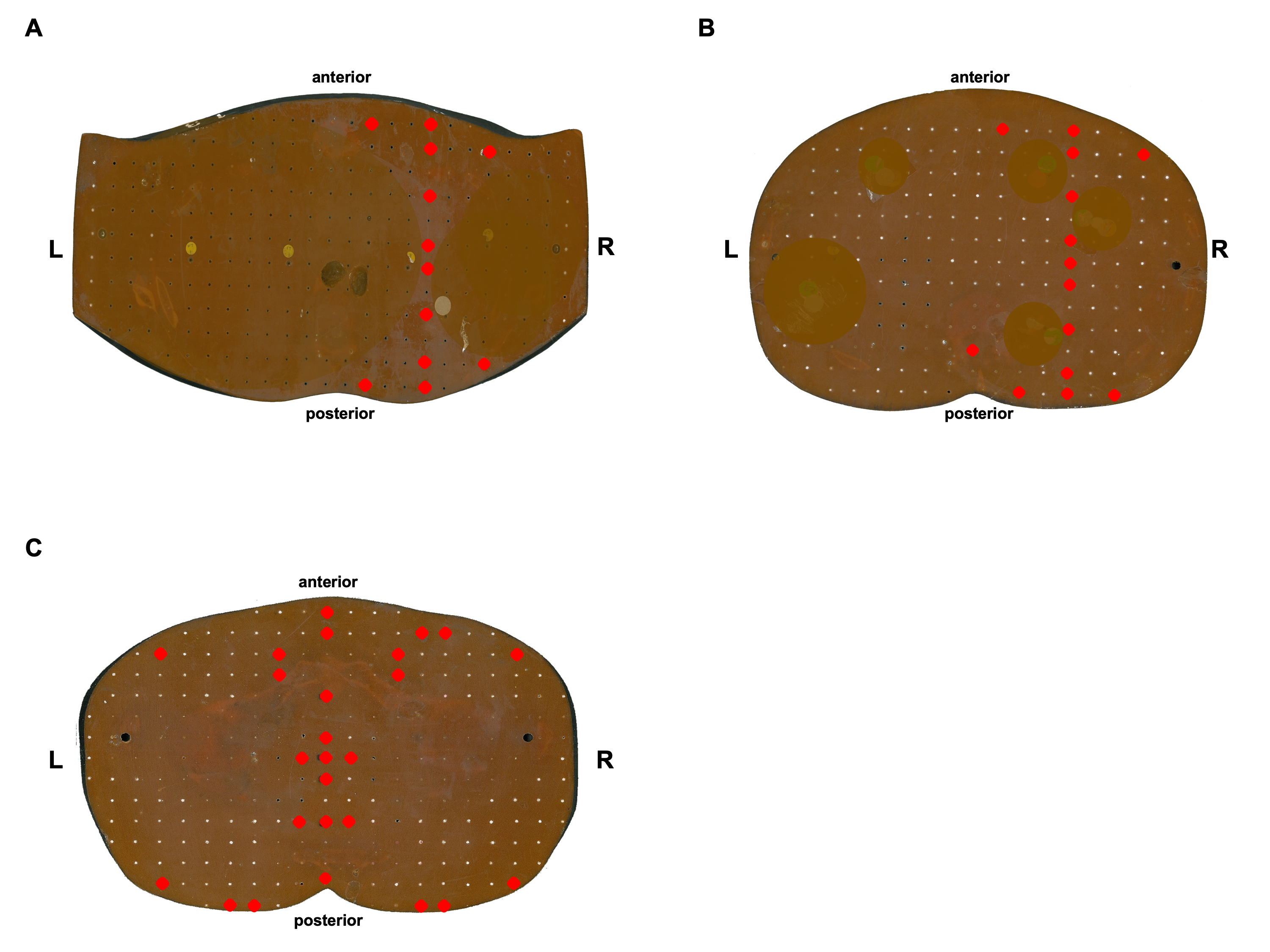

Supplement: Supplementary file 1 [file jcm-13-02732-s001.zip › Supplemental Figure S2.tiff]
